# HIV-1-associated neurocognitive disorder: epidemiology, pathogenesis, diagnosis, and treatment

**DOI:** 10.1007/s00415-017-8503-2

**Published:** 2017-05-31

**Authors:** Christian Eggers, Gabriele Arendt, Katrin Hahn, Ingo W. Husstedt, Matthias Maschke, Eva Neuen-Jacob, Mark Obermann, Thorsten Rosenkranz, Eva Schielke, Elmar Straube, For the German Association of Neuro-AIDS und Neuro-Infectiology (DGNANI)

**Affiliations:** 1 Department of Neurology, Krankenhaus Barmherzige Brüder, Seilerstätte 2, 4021 Linz, Austria; 2 Neurologische Klinik, Universitätsklinikum Düsseldorf, Düsseldorf, Germany; 3 Neurologische Klinik, Charité, Berlin, Germany; 4 Klinik für Neurologie, Universitätsklinikum Münster, Münster, Germany; 5 Neurologische Abteilung, Brüderkrankenhaus Trier, Trier, Germany; 6 Institut für Neuropathologie, Universitätsklinikum Düsseldorf, Düsseldorf, Germany; 7 Direktor des Zentrums für Neurologie, Asklepios Kliniken Schildautal, Seesen, Germany; 8 Neurologische Abteilung, Asklepios-Klinik Hamburg-St. Georg, Hamburg, Germany; 9 Praxis für Neurologie Berlin-Mitte, 10117 Berlin, Germany; 10 HIV-Schwerpunktpraxis, 30890 Barsinghausen, Germany

**Keywords:** HIV-1 infection, AIDS, Neurocognitive disorder, Dementia, HIV-associated neurocognitive disorders (HAND)

## Abstract

The modern antiretroviral treatment of human immunodeficiency virus (HIV-1) infection has considerably lowered the incidence of opportunistic infections. With the exception of the most severe dementia manifestations, the incidence and prevalence of HIV-associated neurocognitive disorders (HAND) have not decreased, and HAND continues to be relevant in daily clinical practice. Now, HAND occurs in earlier stages of HIV infection, and the clinical course differs from that before the widespread use of combination antiretroviral treatment (cART). The predominant clinical feature is a subcortical dementia with deficits in the domains concentration, attention, and memory. Motor signs such as gait disturbance and impaired manual dexterity have become less prominent. Prior to the advent of cART, the cerebral dysfunction could at least partially be explained by the viral load and by virus-associated histopathological findings. In subjects where cART has led to undetectable or at least very low viral load, the pathogenic virus–brain interaction is less direct, and an array of poorly understood immunological and probably toxic phenomena are discussed. This paper gives an overview of the current concepts in the field of HAND and provides suggestions for the diagnostic and therapeutic management.

## Introduction and terminology

Since the introduction of combined antiretroviral therapy (cART) in 1996, the HIV-1 infection has become a treatable condition. However, HIV infection is an incurable disease as attempts to eradicate the virus have so far been unsuccessful. The cerebral manifestations of HIV infection with the disturbance of cognitive, behavioural, motor, and autonomous functions [2, 72, 83, 113] remain an issue in the everyday practice of HIV medicine. The older terms HIV encephalopathy and AIDS dementia complex have been replaced by the term HIV-associated neurocognitive disorder (HAND). The current terminology of HAND [1] is based on a 2007 revision of the older classification of 1991 and was triggered by the fact that the disease course was considerably altered by cART (Table 1). The current terminology of neurocognitive impairment (NCI) comprises the category of asymptomatic neurocognitive impairment (ANI), mild neurocognitive disorder (MND), and HIV-associated dementia (HAD). Motor and psychiatric findings are no longer required for diagnosis. We do, however, still recommend to ascertain motor and affective symptoms and signs, as they are a constitutive elements of HAND and are relatively easily quantifiable irrespective of the patients ethnic and educational background [3, 98]. In a clinical-pathologic study, a good agreement of the histopathological diagnosis of HAND with the new classification scheme was found [12].

**Table 1:** International terminology of HIV-associated neurocognitive disorders (HAND) [1]

HIV-1-associated asymptomatic neurocognitive impairment (ANI)	Acquired impairment in cognitive functioning (NCI), involving at least two ability domains, documented by performance of at least 1,0 SD below the mean^a^ on standardized neuropsychological tests^b^ The cognitive impairment does not interfere with everyday functioning (e.g., mental acuity, inefficiency in work, homemaking, or social functioning)
HIV-1-associated mild neurocognitive disorder (MND)	Neuropsychological test results as with ANI At least mild interference in daily functioning (self-report or witnessed by knowledgeable others)
HIV-1-associated dementia (HAD)	Neuropsychological test results as with ANI, but performance in cognitive testing impaired by at least 2 SD of the mean Marked interference with day-to-day functioning

^a^Adjusted for age-education-appropriate norms

^b^The neuropsychological assessment must survey at least the following abilities: verbal/language; attention/working memory; abstraction/executive; memory (learning; recall); speed of information processing; sensory-perceptual, motor skills

## Epidemiology

Since the introduction of cART in 1996, the incidence of HIV-associated diseases in the industrialised countries has decreased to the extent that the life expectancy of HIV-infected people is now close to that of non-infected individuals [68]. The incidence of HAND was found to be less decreasing than that of the other AIDS-defining conditions [25], although the most severe forms of dementia are much rarer now The prevalence of neurocognitive dysfunction caused by HIV itself (as apposed to opportunistic infections) increases over time after infection and is currently estimated at 20–50% [55, 100, 111]. HAND is a treatable condition, and the treatment effect is larger in cART-naive and more impaired, i.e., demented, patients. Several authors reported patients with suppressed plasma viraemia who developed chronic progressive and at times fluctuating cognitive dysfunction [1, 5, 124]. One longitudinal study of cognitive dysfunction in asymptomatic HIV-infected individuals yielded stable cognitive performance over 5 years [18]. Patients who were diagnosed and treated early after infection had a low prevalence of NCI [19]. HIV patients, however, who started their cART late, i.e., with a low CD4 cell count, and where observed over several years were found to be cognitively somewhat more impaired than HIV-negative individuals [75]. Longitudinal cohort observations found that many patients with asymptomatic neurocognitive impairment (ANI), even with suppressed plasma viral load, eventually developed symptomatic NCI [45]. Although the widespread use of cART has lead to a marked decrease in the number of patients with more severe manifestation of HAND [90], less severe NCI remains frequently being observed in clinical practice, and these manifestations now occur earlier during the course of HIV infection [54, 55, 99, 111].

HAND is associated with shortened survival [105]. In patients with a known time point of the primary HIV infection, an early decrease of CD4 cell count and high initial plasma viraemia were found to predict NCI [69]. Patients in whom the primary HIV infection is clinically manifest have an earlier onset and a more rapid course of NCI [127].

In 2015, 29,747 people were newly diagnosed with HIV in the European Union and European Economic Area, with a rate of 6.3 per 100,000 (http://ECDC.europa.eu; accessed March 2017).

The mean age of people living with HIV is increasing. The highest prevalence is now in the age group around 50 years. This implies that the differential diagnosis of NCI of HIV-infected patients needs to encompass virus-independent and age-associated diseases. The odds of developing HAND increase with rising age [13, 71, 121].

## History and clinical findings

HAND is characterised by an insidious onset with slow progression. If the neurocognitive disorder has an onset of less than 4 weeks competing aetiologies need to be excluded. At the time of clinical and neurocognitive testing, the patient must not be febrile, extremely tired, sedated by medication, or suffer from an acute disease with an impact on his physical and mental condition. In this situation, a repeat clinical and psychometric examination under suitable conditions is required.

In the earlier stages, patients with HAND complain of difficulties in concentration and memory and of impaired executive functions. When the disease progresses, signs of psychomotor slowing with depressive and other affective symptoms such as irritability as well as mild and sometimes subclinical motor signs will be apparent. The full-blown dementia that, if untreated, may progress to a bedridden state with mutism and incontinence is discussed to be a distinct phenomenon with a partially different pathogenesis.

Expressing complaints about mental dysfunction is not the same as actually having objective impairment. Patients in whom neuropsychological testing actually demonstrates NCI tend to underestimate the degree of their dysfunction, while the opposite is true for patients with depression [119]. This is why a history given by informants close to the patient often is very important.

HAND does not reduce the level of consciousness nor does it cause unequivocal focal signs or neck stiffness. Neither are psychiatric manifestations such as illusion and paranoia sufficient to make the diagnosis of HAND. The co-incidence of HAND and affective and schizophrenic psychosis is very low [53]. Some 5–10% of patients with more advanced HAND may develop focal and generalised epileptic seizures [130].

## Ancillary diagnostic procedures

Neurocognitive testing, radiology studies, biochemical analyses (cerebrospinal fluid, CSF), and electrophysiological studies (EEG, somatosensory evoked potentials, SEP) may aid in the diagnosis of HAND. HAND is, however, a clinical diagnosis, and no technical finding in isolation warrants the diagnosis of HAND.

### Neurocognitive testing

Quantitative neurocognitive testing is the most suitable means and the gold standard to ascertain NCI. Testing should include the neuropsychological domains speech, attention/working memory, abstraction/executive function, learning/recall, information processing speed, and motor functions [67]. Table 2 lists some frequently used tests.

**Table 2:** Neuropsychological test methods suitable for the screening and diagnosis of HAND [1]

	Test	Cognitive domain/function
Simple tests (preferred for screening)	HIV dementia scale [7, 78], International HIV dementia scale [101]; MoCA test [58, 86]; “NEU screen” [81]	Working/short-term memory, attention, interference, visuoconstruction
In-depth testing (“gold standard”)	Trail-making, grooved peg board, digit-symbol test, reaction time	Psychomotor speed
	Trail-making, Stroop colour-word-interference test, digit-symbol test	Abstraction
	Copy drawing of Reye figure, mosaic test	Visuoconstruction
	Repeating of multi-digit-numbers, Rey-Auditory-Verbal Learning test, digit-symbol test	Attention and memory

For interpretation, the results are adjusted for age and length of education

Where comprehensive testing is not possible, shorter tests may be applied. The HIV dementia scale features age- and education-adjusted norms [78]. The International HIV dementia (IHDS) scale is validated and independent from education [101]. The Montreal cognitive Assessment (MoCA) test is well established in Alzheimer’s disease and has been applied to diagnose HAND. Their sensitivity and specificity are, however, very limited [58, 77].

Among the electrophysiological tests, the value of the EEG is to differentiate from epilepsy-associated psychoorganic states. In HAND, the EEG is usually normal, but may show slight generalised slowing without focal findings. The finger tapping test is sensitive to motor slowing [2].

Magnetic resonance imaging (MRI) of the brain is the most sensitive imaging modality. It should encompass the following: axial DWI, T2, TIRM/FLAIR, and T1 series; additionally T1 with contrast enhancement (when appropriate) and the T2 or TIRM/FLAIR series in the sagittal plane. MRI often shows hyperintense signal in the deep white matter and in the basal ganglia of the cerebral hemispheres, but this finding is not specific for HAND [117]. As opposed to progressive multifocal leukoencephalopathy (PML), the cortical U-fibres are not involved in HAND. Enlargement of ventricles and the cortical sulci may occur early in the disease course [92]. Space occupying lesions and focal contrast enhancement are incompatible with the diagnosis of HAND. The main use of brain imaging is, however, the exclusion of diseases mimicking HAND.

Modern MR-based techniques such as MR spectroscopy, magnetization transfer ratio (MTR), diffusion tensor imaging (DTI), and voxel-based morphometry have all been shown to correlate structural and biochemical changes with neurocognitive parameters [17, 52, 60, 93], but they are not yet established in clinical routine.

With a large part of the HIV-infected population growing old, HIV-associated MRI changes need to be differentiated from white matter hyperintensities (WMH) associated with cerebral small vessel disease, and both may co-exist. In a study with cART-treated subjects, the extent of the white matter hyperintensities was more closely correlated with the subject’s age and blood pressure than with virological parameters [76].

The value of psychiatric examination lies in the differentiation of HAND from major depressive disorder as depression frequently occurs in HIV infection, and there is an overlap between the symptoms of depression and HAND [125].

Cerebrospinal fluid (CSF) is mainly taken to diagnose CNS opportunistic infections and CNS lymphoma (Table 3). In patients with HAND, the CSF white cell count may be normal but frequently shows a mononuclear pleocytosis of up to 20 cells/µl. With severe immunosuppression, it may be decreased. Total protein and albumin concentrations may be slightly elevated (blood–brain barrier disruption). Oligoclonal bands and increased IgG-index indicate autochthonous immunoglobulin production within the CNS. However, these findings, including slight pleocytosis, are non-specific and are frequently present even in the asymptomatic stages of HIV infection [35]. In patients on cART, the CSF cell count is lower than in untreated subjects, and this applies especially to cART regimens with CSF penetrating substances [70]. A CSF pleocytosis that arises within weeks after initiation of cART may suggest an immunological response to HIV in the context of immune reconstitution. In untreated patients, there is a weak but statistically significant correlation of (higher) CSF viral load with HAND [6, 73]. However, this association is no longer true for individuals on cART [21, 55, 74, 104]. In CNS opportunistic infections, there is an elevated HIV RNA concentration in the CSF, thereby restricting the use of HIV viral load in the CSF for the diagnosis of HAND [79].

**Table 3:** Differential diagnoses of HAND and the relevant diagnostic procedures

Disease	Useful diagnostic procedures (commentary)
Primary and degenerative dementias Cerebral small vessel disease, Alzheimer, Lewy-body-dementia, frontotemporal dementia, normal pressure hydrozephalus, and Parkinson’s disease	History (typical pattern of clinical presentation?) including family history and arterial hypertension with end organ damage Thorough neurological examination Neuropsychological-cognitive profile Routine MRI, FDG-PET CSF including “biomarkers” such as Aβ42 and tau CSF opening pressure and, if applicable, drainage of 20–30 ml CSF
Creutzfeld–Jakob disease	14-3-3-protein and *tau* in the CSF, EEG, MRI
Cognitive dysfunction with concomitant major depressive disorder (“pseudodementia”)	Psychiatric examination
Intoxication	Determination of prescription and illicit drugs in plasma and urine
Progressive multifocal leukoencephalopathy (PML)	MRI with white matter lesions (Gadolinium enhancement may occur with immune reconstitution inflammatory syndrome (IRIS)) PCR for JC virus in the CSF
Metabolic encephalopathy and poor general condition	Blood chemistry (electrolytes, kidney, liver function tests, thyroid stimul. hormone (TSH) cortisol, differential blood count) Vitamin-B_12_ deficiency (homocysteine and holo-transcobalamine in the serum) Hypoxaemia? (blood gas analysis) Poor general condition? (bed ridden, cachexia, pyrexia)
Neurosyphilis	Antibody testing including CSF analysis
Primary CNS lymphoma	CT/MRI/PET or SPECT (uni- or multifocal lesions, mostly located close to the ventricles; reduced diffusion on diffusion-weighted imaging)
Toxoplasmosis	CT/MRI (uni- or multifocal contrast enhancing lesions; enhanced diffusion in the lesion core on diffusion-weighted imaging) Antibody testing in blood and CSF (absence of any antibody makes toxoplasmosis unlikely)
CMV encephalitis	CSF including CMV-PCR; pp65 antigen in blood, antibody testing for CMV; MRI (subependymal contrast enhancement); fundoscopy for CMV retinitis
Cryptococcosis	CSF including opening pressure, ink stain, fungal culture, cryptococcal antigen in CSF
VZV encephalitis	CSF including PCR for varicella zoster virus and antibody testing
Tuberculous meningitis/other bacterial agents	CSF including Gram staining, bacterial culture (including Tb), DNA-based methods IFN-gamma release assays, Tine test and Mendell–Mantoux test may all be negative due to immunosuppression
Hepatitis-C virus infection	Antibody testing, PCR, and liver function tests
Immune reconstitution inflammatory syndrome (IRIS)	Well known for Tb, PML, cryptococcosis, and toxoplasmosis

CSF levels of Aβ42 (a cleavage product of the amyloid precursor protein, APP) and the protein τ (tau) have been found to be decreased and increased, respectively, in HAND patients [15, 115]. The patient populations were small, however, and these parameters are not yet recommended for clinical routine. Even in patients with suppressed plasma virus, neopterin as a marker of immune activation and neurofilament-light as a marker of tissue damage are elevated in the CSF and correlate with NCI [26].

## Differential diagnoses

In view of an aging HIV-population, a declining incidence of CNS opportunistic infections, and HIV infection being a vascular risk factor, other aetiologies of neurocognitive dysfunction need to be considered. These include non-HIV-associated conditions such as Alzheimer’s disease, cerebral small vessel disease with vascular dementia, dementia with Lewy bodies, communicating hydrocephalus, etc. Table 3 lists the most important differential diagnoses and the appropriate clinical work-up.

## Pathogenesis and neuropathological findings

In patients with no or an unsuccessful antiviral treatment, the morphological hallmark of HAND is HIV encephalitis and HIV leukoencephalopathy. The encephalitis is characterised by disseminated infiltrates of lymphocytes, macrophages, and multinucleated giant cells [8], while leukoencephalopathy implies bilateral diffuse loss of myelin in the hemispheric white matter alongside with astrocytosis and microglial infiltration [9]. The most typical (albeit not pathognomonic) finding is the multinucleated giant cells, which arise from fusion of macrophages.

During the course of primary infection, HIV enters the brain parenchyma via infected lymphocytes and monocytes and probably by transependymal migration [48]. This is the reason why inflammatory CSF changes are already present in the asymptomatic stages of the infection in almost all individuals [35, 70]. The basal ganglia and the frontal white matter are the most early and intensely affected brain regions [84]. The cellular basis of carriage and production of virus are the immunocompetent cells such as perivascular microglia and lymphocytes [106]. Neurons, astrocytes, and oligodendroglia are not or only minimally infected. They are, however, affected as evidenced by loss of synapses and neurons [38, 47], by apoptosis as well as by production of osteopontin, a pro-inflammatory cytokine [109]. Many authors found a positive correlation between the amount of virus and viral products (e.g., gp120 und gp41) and the extent of histopathologic changes in the brain [24, 66, 87, 118, 128]. In addition, the grade of clinical neurologic dysfunction is related to the extent of macrophage activation on histopathology [14, 24, 43]. A higher CSF viral load predicts the future development of HAND [34]. Although the causality scheme *virus/viral products* → *histopathologic changes* → *neurocognitive dysfunction* is well established with statistical significance, this relationship is not very close. Even in the pre-cART era, the CSF viral load was only slightly higher in HAND subjects vs. non-HAND subjects, and this relation is no more true in the cART era [21, 55, 74, 104].

Infact, in patients on plasma-suppressive cART, despite suffering from HAND, the classical features of HIV encephalitis often are conspicuously absent [37]. Here, metabolic effects, e.g., at the blood–brain interface, and functional changes rather than irreversible structural damage are being discussed as pathogenic factors [42].

Thus, HAND is not caused by a one-dimensional and direct pathogenetic event, but rather by multi-dimensional and complex immunopathological processes that are governed by viral as well as host factors.

With its high genetic variability, HIV rapidly adapts to the cellular and immunologic environment. Early isolated findings on brain-specific genetic HIV sequences could later not be replicated [56]. However, many authors found genetic diversity of CNS virus as compared to virus from systemic compartments such as blood, spleen, and bone marrow, and this difference was more pronounced in HAND vs. non-HAND patients [29, 57, 116]. Multiple studies found an at least partial compartmentalisation of virus replication in the CNS and blood, and this was interpreted as evidence for adaptation to organ specific environments [28, 94].

A variety of cytokines and chemokines involved in cellular and humoral immune processes are correlated to the grade of brain infection [85, 114]. The chemokine CCL-2, e.g., that governs the invasion of immunocompetent cells into the CSF and stimulates virus replication, is associated with neurocognitive function [104, 131]. Several authors found specific genotypes (involving, e.g., CCL-2, TNF-alpha, and the Delta-32-deletion of the CCR5 molecule) to predispose to the development of HAND [44, 91, 122].

While in the pre-cART era, the CD4 cell count and the plasma viral load predicted the development of HAND, this is no more true for the cART era. In addition, HAND does now occur in earlier stages of the HIV infection [55]. Several studies have identified the following risk factors for developing HAND: lower education, severe pre-existing immunosuppression, older age, history of AIDS-defining illnesses, high TNF-alpha and MCP-1 plasma concentrations and, most prominently, lower nadir CD4 count [4, 32, 96, 104, 120]. An international study with, on average, 40-year-old cART-treated participants found an association of cardiovascular diseases and their risk factors (blood pressure and cholesterol) with worse neurocognitive function [132]. For the phenomenon of development and persistence of HAND despite desirable systemic viral and immunologic parameters, several explanations have been proposed. Some studies in patients with long-term suppressed virus load in the plasma and CSF found elevated levels of neopterin and anti-MOG antibodies in the CSF [27, 61] and microglial activation on brain positron emission tomography (PET) [40]. This was interpreted as chronic immune activation in the CNS. The dissociation of immunologic events in the CNS from the systemic compartment may suggest persistent low-level virus replication in the brain. In fact, low-level virus production has been found in the CSF of patients by the use of an extra-sensitive PCR protocol detecting down to 2 copies/ml [65].

Nowadays, the most severe manifestations of HAND do almost only affect either untreated or insufficiently treated subjects [37, 90].

Despite the brain infection taking place in the days after primary infection, the development of HAND takes years. As an explanation for this ostensible contradiction, it has been suggested that initially, the brain infection is relatively well controlled, while later, there is a quantitative and qualitative breakdown of immune control in the CNS [89].

Studies showing high copy numbers of HIV DNA in mononuclear cells including monocytes in HAND patients [59, 107] as well as studies showing high numbers of circulating monocytes in children with HAND [102] suggest a role of macrophages in the pathogenesis. The pathogenic role of the immune response is supported by the phenomenon of the immune reconstitution inflammatory syndrome (IRIS). This may be characterised by marked leukoencephalopathy and an unusual infiltration of CD8-positive lymphocytes [46, 124].

Findings of amyloid deposition in the brain parenchyma and pathologically low or high values of Aβ42 and the axonal protein *tau*, respectively, in the CSF, point to aspects of the Alzheimer’s disease pathology [15, 37, 49].

While some authors implicated HCV co-infection in the pathogenesis of HAND, a recent large and well-controlled study found no evidence for worse cognitive function in HCV co-infected patients, at least in the absence of liver dysfunction [16].

Surprisingly, one study showed improved cognitive function in patients during interruption of long-term and suppressive cART [97]. One possible explanation is mitochondrial toxicity of cART that has been suggested by MR spectroscopy showing reduced levels of the mitochondrial and neuron-associated molecule *N*-acetylaspartate (NAA) in individuals treated with d4t or ddI [103].

## Treatment and prevention

With the assumption that the HIV infection of the brain is the necessary prerequisite for the development of HAND, the mainstay of a causal treatment is the suppression of virus replication in the brain. In treatment-naïve patients, various studies employing different methodologies have demonstrated this to be an achievable goal. cART leads to a lower virus load in the brain parenchyma as well as in the CSF, there is improvement of electrophysiologic parameters and, finally, a randomised clinical study has demonstrated improved cognitive function [30, 31, 39, 62, 108, 126]. Notable improvement starts some 4–8 months after start of treatment [22]. The degree of clinical improvement is higher in more severely affected patients, and it corresponds to the increase of CD4-lymphocytes [54, 55].

While it is fully accepted that untreated patients with HAND need to be started on cART, the question of how to treat is less clear with patients already on cART. If there still is ongoing plasma virus replication, this should obviously be suppressed by adapting the cART. If plasma virus is suppressed lumbar puncture for the determination of CSF viral load, and if possible, drug-resistant virus should be done. For the detection of CSF virus, an ultrasensitive PCR technique detecting down to 2 copies per ml CSF might be employed, as low-level virus replication was shown to be associated with NCI [65]. In case of ongoing CSF virus replication, but also without, further modification into “neuro-active” cART may be reasonable. At this stage, differential diagnoses of HAND also need to be considered (Table 3). The MIND EXCHANGE working group suggested a useful algorithm [77]. The issue of which substances and which combination thereof are best suited for the treatment and prevention of HAND is still under debate. Conventional wisdom suggests an important role of the penetration of antiviral substances into the CSF and brain parenchyma. This is supported by a randomised trial that compared a protease inhibitor (PI) monotherapy (lopinavir, LPV/r) to a dual cART with LPV/r and nucleoside/nucleotide analogues [51]. There was detectable viral load in the CSF with suppressed or very low viral load in the plasma in some of the monotherapy patients.

A CNS penetration effectiveness score (CPE), composed of the relative values of CNS penetration of the substances in a given cART regimen, has been devised by Letendre [63]. It comprises four categories, where lower scores indicate lower CNS penetration (Table 4).

**Table 4:** CNS penetration effectiveness score (CPE) [63], updated according to Letendre 2014

	4	3	2	1
NRTI’s	Zidovudine	Abacavir Emtricitabine	Didanosine Lamivudine Stavudine	Tenofovir
NNRTI’s	Nevirapine	Efavirenz Etravirine	Rilpivirine	
PI’s	Indinavir/r	Darunavir/r Fosamprenavir/r Indinavir Lopinavir/r	Atazanavir Atazanavir/r Fosamprenavir	Nelfinavir Ritonavir Saquinavir Saquinavir/r Tipranavir/r
Entry/fusion inhibitors		Maraviroc		Enfuvirtide
Integrase inhibitors	Dolutegravir	Raltegravir	Elvitegravir	

A value of 1, 2, 3, or 4 is assigned to the different antiviral substances (first line). The CPE values of a patient’s cART components are summed up to arrive at the CPE score. A high score stands for better penetration into the CNS

Several authors worked on the impact of the CNS penetration on the CSF viral load and on cognitive clinical endpoints. Most studies showed higher CPE scores to be associated with lower CSF viral loads [20, 23, 64]. Whether this translates into neurocognitive improvement is less clear. A review of observational studies and two more recent observational studies showed only moderate but statistically significant desirable effects [11, 23, 123]. Two small randomized trials prospectively examined cART regimens with higher vs. lower CPE scores. One showed slightly better cognitive results with higher CPE scores [129], and in the other, there was a trend for such an effect in the whole study population and a statistically significant positive effect in the subgroup with suppressed plasma viraemia [33]. One randomised trial tested Maraviroc vs. Tenofovir against the background of Darunavir and Emtricitabine in treatment-naïve patients. There was cognitive improvement in both arms but no difference between the two agents [95].

In a small randomised, controlled pilot trial of cART-intensification with Maraviroc on the background of stable and plasma-suppressive cART, Maraviroc significantly improved cognition [41].

Thus, there is no final proof of better neurocognitive performance with CNS-penetrating antivirals. Nevertheless, at least in patients with symptomatic CNS disease, we recommend to take the CPE score into consideration.

The notion of the importance of suppressing the CNS viral replication is supported by case series that describe patients with long-standing suppression of the plasma viral load but detectable viral replication in the CSF (“viral escape”) and cases with resistant viral strains in the CSF [10, 88, 112]. These subjects had clinically overt neurological disease, and on optimization of their cART according to the CPE score and resistance testing, all improved clinically and in terms of CSF viral load.

The European AIDS Clinical Society in its latest guideline (Oct 2016) recommends to screen for and, if appropriate, to perform the diagnostic steps for HAND. In case of established diagnosis, considering CNS-active drugs is recommended (http://www.eacsociety.org/guidelines/). The US Department of Health and Human Services (HHS), in its latest guideline (July 2016), recommends to consider CNS pharmacokinetics only in the case of clear-cut CNS viral escape with clinical manifestations (https://aidsinfo.nih.gov/guidelines/).

In the differential diagnosis of NCI, toxic effects of cART substances need to be considered. Efavirenz is well known to cause neuropsychiatric symptoms, but these tend to wear off with time. Some authors found NCI in patients on stable cART that improved on discontinuation of cART [50, 80, 97], although others failed to replicate these findings. Although structured therapeutic interruptions (STI) arise as a plausible option, these are not recommended as they may cause more harm than benefit. If neurotoxicity is suspected, modification of the cART may be reasonable.

A variety of non-antiretroviral substances such as minocycline, memantine, selegiline, lithium, valproate, lexipafant, nimodipine, psychostimulants, rivastigmine, and others have been tested for the treatment of HAND. None of these exerted a significant clinically beneficial effect [82, 110].

Non-pharmacological interventions that may have a positive clinical effect are the treatment of concurrent diseases such as HCV infection with liver dysfunction, major depressive disorder, and cardiovascular and metabolic risk factors, as well as improving the adherence to cART [36].

Some groups advocate screening for HAND, while others are hesitant as there is no scientifically established concept of reacting to pathologic results of screening. Moreover, the comprehensive neurocognitive test battery (the gold standard) is time-consuming and not widely available. There are shorter tests such as the HIV dementia scale [78], the MoCA test [7, 58, 86], and the NEU screen (a combination of the trail-making test A and B with a verbal-learning test) [81], but they have limited sensitivity and specificity. If screening is decided, it should be done early after the diagnosis of HIV infection and preferentially prior the commencement of cART as this provides baseline data for longitudinal testing [77]. According to the individual risk constellation, the interval of the screening examinations should be 6–24 months.

The most prominent cognitive feature differentiating HAND from Alzheimer’s disease is the memory loss in Alzheimer’s which affects encoding as well as retrieval.

For the prevention of HAND, an early start of cART might be beneficial, although there are no randomised studies [19, 32]. The value of CNS-penetrating substances for the prevention of HAND (as opposed to its treatment) is not established.

## Outlook

Considering its treatability, the continuously high number of yearly transmissions and of migrants from endemic regions, the prevalence of HIV infection is bound to increase. The rising age of the HIV-infected population will make it ever more necessary to include non-HIV-associated forms of dementia into the differential diagnoses of HAND. As not all HIV patients will eventually suffer from HAND, specific parameters allowing for risk stratification would be helpful. This includes better instruments for screening and diagnosis. Because, for the time being, HIV cannot be eradicated from the body improved cART substances as well as other principles of preventing and treating HAND are needed.

## Practical conclusion

- The prevalence of HIV infection and the age of the infected population are rising.
- HAND is a subcortical type of cognitive disease with psychomotor slowing as the most salient feature.
- The differentiation from other types of cognitive disorders becomes ever more important.
- The diagnosis of HAND is made on clinical grounds. Ancillary diagnostics steps are done to exclude differential diagnoses.
- HIV patients should be regularly screened for neurocognitive dysfunction.
- For treatment of HAND, CNS-penetrating substances should be considered.
